# Experiences and Perceptions of Registered Nurses Who Work in Acute Care Regarding Incident Reporting: A Scoping Review

**DOI:** 10.3390/healthcare13111250

**Published:** 2025-05-26

**Authors:** Clara Smit, Monica Peddle

**Affiliations:** 1 Intensive Care Unit, St Vincent’s Hospital Melbourne, Fitzroy, VIC 3065, Australia; 2 Faculty of Health, School of Nursing and Midwifery, Deakin University, Burwood, VIC 3125, Australia; m.peddle@deakin.edu.au

**Keywords:** incident reports, nursing staff, hospitals, patient safety, just culture

## Abstract

**Background/Objectives**: Clinical incidents can be valuable learning tools to improve patient safety. However, failure to report or underreporting of clinical incidents is a global phenomenon. Understanding nurses’ experiences is essential to identifying challenges and developing strategies to enhance incident reporting behaviours. This review aimed to explore the experiences and perceptions of acute care bedside nurses regarding incident reporting. **Methods**: This review used scoping review methods. A search of the MEDLINE and CINAHL databases returned 16 papers that were included in the review. **Results**: Five main themes were identified—Fear of Reporting, Levels of Reporting, Lack of Knowledge, Education and Training on Reporting, Benefits of Reporting, and Changing the Culture. **Conclusions**: Nurses experience fear of incident reporting stemming from negative repercussions and the organisational blame culture. Lack of knowledge and training about errors and incident reporting processes limits incident reporting behaviours. To enhance reporting behaviours, promoting a just culture that includes the support of managers, open communication, and feedback on incidents is important. Education and training can also enhance nurses’ awareness and capability of incident reporting.

## 1. Background

Unsafe care is estimated to cause over three million deaths every year worldwide [1]. A clinical incident is any unintended event or unsafe condition resulting from the care process, with patient outcomes ranging from near-misses to incidents resulting in severe harm or death [2]. An estimated 42.7 million patients worldwide experience adverse events when in hospital [3]. Clinical incidents and adverse events can significantly impact patients, healthcare organisations, and healthcare workers [4]. However, a significant gap remains in understanding the precise series of events and the system weaknesses that lead to safety incidents [3].

Despite the potential for damaging consequences of patient harm, clinical incidents can be helpful learning tools to improve patient safety if they are reported [5]. Incident reporting is designed to identify system failures with the information used to implement measures to improve patient safety [6]. Clinicians have an ethical and moral responsibility to provide safe care, act in the patient’s best interest, be open and honest about errors or near-misses, and commit to learning from mistakes [7]. While incident reporting is considered an effective method for improving healthcare safety, healthcare organisations and clinicians often overlook the actual reporting of incidents [8].

Failure to report or underreporting of incidents is a global phenomenon and a significant hurdle in patient safety [9]. Yung et al. [10] indicate that the rate of error reporting in the United Kingdom is 22–39%, in Taiwan it is 30–48%, and in the United States of America it is 40–50%. Some studies have estimated that clinicians report clinical incidents with a frequency between 1% and 3% of total cases [11,12,13]. Research has been conducted to explore the barriers to incident reporting, with findings suggesting inadequate incident reporting systems, poor managerial conduct, lack of multidisciplinary collaboration, and limited training as core elements [14]. Inadequate incident reporting systems are the absence of organisational support required to foster incident reporting behaviours in nurses, such as policies and effective interventions to promote reporting [14]. One such organisational intervention in promoting incident reporting is the establishment of a “just culture”, which has been shown to increase the number of reported patient safety incidents, help staff learn from incidents, and reduce the risk of further patient safety incidents [15]. A just culture encourages healthcare workers to speak up about patient safety, does not apply blame but rewards staff for doing so, and ultimately increases trust among healthcare staff [15].

Bedside nurses are direct providers of care and, therefore, have an essential role in keeping patients safe [16]. Additionally, the high-stress, fast-paced nature of acute care nursing exacerbates the risk of errors, making nurses particularly vulnerable to mistakes and near-misses [17]. The evidence outlined above indicates that there are barriers that contribute to failure to report and significant underreporting. However, it is also important to understand the perspectives of nurses in incident reporting, as bedside nurses often act as the first line of defence to prevent errors for hospitalised patients [17]. Understanding bedside nurses’ perspectives and experiences with incident reporting is essential to identifying their challenges and developing suitable strategies to assist in improving nurses’ reporting practices and patient safety.

## 2. Aim

This scoping review aimed to answer the following research question: What are the experiences and perceptions of acute care bedside nurses regarding incident reporting?

## 3. Methods

A scoping review was deemed suitable as the review aims to map the evidence regarding bedside nurses’ experiences and perceptions of completing incident reports in acute care settings. The findings from the review will assist in identifying the gaps in knowledge and inform future research. This scoping review is conducted in adherence to the Updated Methodological Guidance for the Conduct of Scoping Reviews [18], guided by a protocol and reported using the PRISMA extension for Scoping Reviews (PRISMA-Scr) [19]. We did not register a protocol for this review as this is not mandatory for a scoping review. However, a protocol was developed to guide the review, detailing the scope, search strategy, inclusion and exclusion criteria, and data extraction approach. The research question was formulated using the population, phenomenon of interest, and context structure (PPIC). Following this, the JBI Manual for Evidence Synthesis guided the scoping review [20].

### 3.1. Search Strategy

Articles from January 2019 to December 2024 were identified by searching MEDLINE in the EBSCOhost database. The subject headings “nursing staff, hospital”, “incident reports”, and “hospitals+” were used in the search. The following search terms were searched for in the title and abstracts of articles: “nurs*”, “experience*”, “perception*”, “attitude*”, “view*”, “feeling*”, “perspective*”, “hospital*”, or “acute care”. The following terms were searched in the titles and abstracts of articles using proximity searching with the term “report*”: “Incident*” or “error*” or “near-miss*” or “adverse event*”. Search results were limited to English language results. A search using the same keywords and Boolean operators was constructed in CINAHL. Table 1 depicts the search strings used in each database for this scoping review. Initially, 254 articles were returned from MEDLINE, and 197 articles were returned from CINAHL before data reduction commenced.

### 3.2. Inclusion Criteria

Eligible for inclusion were articles reporting (i) empirical studies that (ii) described the experiences and perceptions, (iii) of participants who were nurses employed in acute care hospitals and provided direct patient care, (iv) that documented the reporting of clinical incidents, near-misses, or adverse events, and (vi) were written in English. Only the data relevant to nurses was included in this scoping review. Articles that included other health professionals, such as medical doctors and allied health staff, in their studies alongside nurses were included in the literature review, where the data regarding the nurses were reported separately and could be extracted for analysis.

### 3.3. Exclusion Criteria

Articles were excluded if they focused exclusively on the experiences of nurse managers or clinical leaders, as the focus was on bedside nurses’ reporting. Articles were excluded if they focused on the reporting of incidents of colleagues. Articles that aimed to develop theories or models to predict nurses’ intent to report clinical incidents were excluded, and articles where the data regarding nurses’ experiences and perceptions could not be separated from other health professionals were also omitted. All types of reviews and meta-analyses were excluded to avoid duplication of results.

### 3.4. Data Selection

Titles and abstracts of retrieved papers from each database were screened according to the inclusion and exclusion criteria. Duplicates were screened through EndNote before full-text screening commenced. Two researchers undertook full-text screening, with any conflicts resolved through consensus.

### 3.5. Data Extraction

Relevant study data were extracted and tabulated using the column headings author/publication year, study design, research aim/question, location/setting, participants, and findings. The data extraction table was piloted with the data extracted from Abdelmaksoud et al. [21], the first paper included in the study. Following the pilot, the headings for the overall findings category were updated to include the column headings of Experiences and perceptions of reporting, Incident reporting practices, Barriers and Enablers to reporting, and Recommendations to reflect the work of Abdelmaksoud et al. [21]. Two researchers undertook data extraction of included papers, with any conflicts resolved through discussion and consensus. See Table 2 for characteristics and findings of included studies.

### 3.6. Data Analysis

Microsoft Excel was used to tabulate the data and to support data analysis and organisation. Thematic analysis was used to analyse the extracted data to find common themes in the findings. The researchers read and reread the extracted data to familiarise themselves with it. Patterns and similarities in the data were noted and assigned codes. Codes that were similar were grouped into themes. Data were reviewed in each theme for consistency, coherence, and fit. Themes were named and defined and are reported below.

### 3.7. Quality Appraisal

While quality appraisal is optional in a scoping review, it was conducted in this review to explore the quality of the research being conducted on the topic. No study was excluded based on the quality appraisal score. The quality of evidence was assessed using the JBI critical appraisal tools. A total score for each tool was calculated by identifying elements of the checklist reported in the paper, compared with those absent, and calculated as a percentage of the total number of elements. Each quality appraisal tool used was selected to align with the study design of each included article. The quantitative studies were assessed using the JBI critical appraisal checklist for analytical cross-sectional studies [24]. The qualitative studies were assessed using the JBI Critical Appraisal Checklist for Qualitative Research [22]. Evidence was appraised for its relevance, reliability, validity, and credibility.

## 4. Results

**Figure 1:**
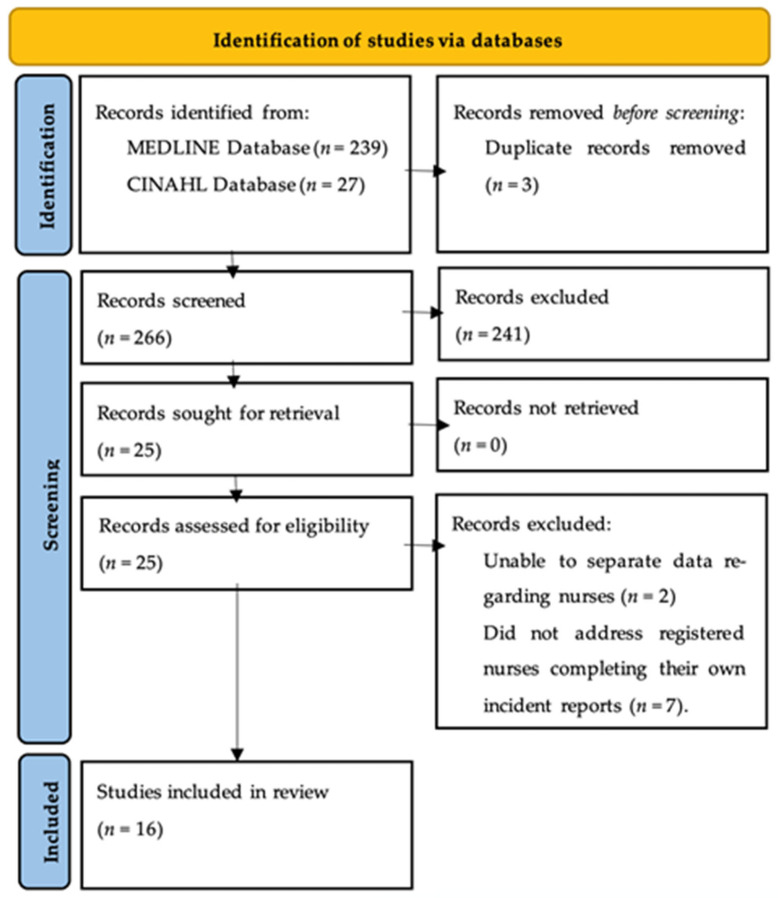
PRISMA_ScR flow chart.

The MEDLINE database search retrieved 239 records, and the CINAHL database returned 27 records. Three duplicates were removed using EndNote. Following a title and abstract review, 25 records were deemed suitable to progress to full-text review. After full-text review, 16 articles met the inclusion criteria and were included in the final scoping review. See Figure 1 for the PRISMA-Scr flowchart [34].

### 4.1. Characteristics of the Included Studies

Studies originated from Iran (*n* = 4), Jordan (*n* = 2), and Saudi Arabia (*n* = 2), with one paper reported each from Australia, Ethiopia, India, Italy, Taiwan, Malta, Palestine, and South Korea (*n* = 1) (Table 2). Thirteen studies used a quantitative research methodology, with 12 authors implementing a cross-sectional research design [2,3,4,7,23,25,26,27,29,31,32,33] and one using an exploratory survey [8]. Three studies used a qualitative methodology, with two authors reporting individual interviews [21,30] and the other using a combination of individual interviews and focus groups [28]. The sample size reported in the papers included in the review totaled 3,782 nurses. In one study, the sample size of nurses compared to other participants could not be determined [7].

### 4.2. Quality of Evidence

All quantitative studies scored 83% on The JBI Critical Appraisal Checklist for Analytical Cross-Sectional Studies (Table 2) [24]. Most studies employed validated surveys and questionnaires, and applied appropriate statistical analysis. The quantitative studies were vulnerable to social desirability response bias, as studies relied on surveys completed by clinicians, which may have led to an overestimation of positive attitudes and desired behaviours [33].

The qualitative studies scored 75–90% on the JBI Critical Appraisal Checklist for Qualitative Research (Table 2) [22]. All the qualitative studies shared a congruent research methodology, research objectives, and the methods used to collect data [22]. As these studies were interested in nurses’ experiences with incident reporting, interviews were the most common way to collect this data. Most qualitative studies presented illustrations from the data to show the basis of the results and ensure that participants are represented [22]. However, it is worth noting that most qualitative articles did not locate the researcher culturally or theoretically and did not address the researcher’s influence on the research [22]. Knowing the researcher’s cultural or theoretical orientation in qualitative studies is important for appraising the evidence because of the researcher’s significant role in the qualitative research process [22]. A statement on the researchers’ reflexivity process and how their assumptions, beliefs, or biases could have influenced the data would have further strengthened the quality of the evidence in these qualitative reviews.

### 4.3. Thematic Results

Five main themes were identified: Fear of Reporting, Levels of Reporting, Lack of Knowledge, Education and Training on Reporting, Benefits of Reporting, and Changing the Culture.

### 4.4. Fear of Reporting

The theme “Fear of Reporting” describes the participants’ overriding feelings toward completing incident reports. Nurses report fearing the consequences of making a report, the fear of being blamed by coworkers, and the fear of the blame culture that pervades clinical practice.

Many nurses feared completing an incident report [8,21,28,31,33]. Fear stemmed from the fear of repercussions [21], including disciplinary action [23,26] and being reprimanded [31]. Nurses reported a fear of demotion and financial penalties [4] and a concern that they are risking their job security [8,28,31,33]. Fear of legal action was another factor reported by nurses [29]. This fear is associated with mistrust, resignation, and scepticism [8]. Additionally, respondents in the study conducted by Al-Oweidat et al. [23] felt uneasy about who else could see the information disclosed in reports.

Some nurses reported being blamed by coworkers when an error occurred (53%), with 44% highlighting unsupportive colleagues [26]. In contrast, while 53% of nurses felt that reporting an error can embarrass a coworker, only 22% (*n* = 31) of nurses indicated that those who made an error were subjected to humiliation or blamed by their colleagues, 25% (*n* = 34) [26]. Nurses were concerned that reporting an incident would result in a change to their social status [28], that they would lose the respect of their colleagues [33], that people would turn against them [3], or because of the reaction of their coworkers manager and executive [32].

Blame culture prevalent in the studied hospitals was identified by 68% of respondents [33]. Additionally, organisational blame culture was described as a barrier to reporting by Abdelmaksoud et al. [21], Mahdaviazad et al. [31], and Napoli [8]. The blame culture emphasised fear of blame from management, lack of governance [28], and poor communication [30] rather than a focus on patient safety [4]. Incident reporting is often perceived to be about disciplining nurses who make mistakes rather than a tool for learning and preventing future errors [32]. Mansouri et al. [4] reported an absence of support from clinical leaders. Conversely, some nurses in the study by Rashed and Hamdan [33] felt supervisors supported those who reported errors. Ward and Mangion [3] observed that respondents considered that reporting adverse events would not result in system improvements that would protect patient safety.

### 4.5. Levels of Reporting

The theme “Levels of reporting” describes nurses’ reporting practices, including their intention to report versus their reporting behaviours and responsibilities.

Discrepancies exist between nurses’ intention to report and the reporting of incidents [23]. Rashed and Hamdan [33] indicated a high level of nurses’ awareness in reporting incidents, and Kapil and Anoopit [29] reported that 80% of nurses were positive about reporting. Similarly, another study stated that 68% of nurses agreed to report errors, 65% felt the need to reveal errors, and 84% indicated that they would not hide or deny reporting errors in self-interest [26]. However, in a Taiwan study, 60% of nurses do not have a voluntary attitude when reporting errors, with only 49% committed to voluntary reporting [27]. Moreover, in the study by Mansouri et al. [4], while 49% (*n* = 123) of the nurses had experienced an adverse event, 71% (*n* = 89) had not reported it. Likewise, in Italy, 43% (*n* = 53) of nurses rarely reported an event, while only 14% (*n* = 17) reported any event witnessed [8]. In Ethiopia, only 37% of nurses reported yes to incident reporting behaviours [2], and some nurses indicated they had a natural inclination to cover up errors [28].

More than half (57%) of nurses in the study by Alsulami et al. [25] agreed that reporting medication errors is their responsibility. However, nurses in other studies were unclear about who was responsible for reporting an incident and to whom the report needed to be made [30]. In the study by Shemsu et al. [2], most nurses, 65%, were uncertain of their obligation to report incidents.

### 4.6. Lack of Knowledge—What to Report

The theme “Lack of knowledge” illustrates the nurse’s knowledge and understanding of what defines an error to be reported. This includes the severity of the error to determine reporting, the knowledge of reporting procedures, and how near-misses are classified.

Knowledge of reportable error definition among nurses was noted as inconsistent [21]. Similar findings were reported by Mahdaviazad et al. [31], with nurses having varied knowledge about the definition, classification, and identification of errors. This ranged from having no idea to knowing the scientific definition, classification, and identification. In a study conducted in Jordan, 47% of nurses were not confident about what constitutes medication errors, and 49% were not sure when to report medication errors using incident reports [32]. Similarly, Kapil and Anoopit [29] identify that 58% of staff had average knowledge of reporting practices. Moreover, Mrayyan et al. [32] indicate that 53% of nurses were confident about what constitutes an error, and 51% were sure when to report errors. This finding is clarified by Mahdaviazad et al. [31], with only 10% of nurses reporting a good understanding of adverse events. A lack of knowledge meant some nurses did not recognise the seriousness of errors and did not report them [30].

In two studies, the seriousness of the error was considered grounds for reporting. The study by Abdelmaksoud et al. [21] clarified that nurses thought the seriousness of an error was the determining factor for lodging an incident report. Similarly, Mrayyan et al. [32] reported that 52% of nurses considered medication errors must be “serious” to be reported. Conversely, findings from Alsulami et al. [25] indicated that regardless of the seriousness of the incident, nurses favoured reporting.

### 4.7. Lack of Knowledge—How to Report

There is variation in the understanding of reporting procedures, with many nurses having a basic knowledge of these procedures [21,29]. In Ethiopia, 43% of nurses did not know where to find an incident form or how to lodge it [2]. In Italy, only 40% of nurses in the study were aware of the online reporting system [8]. In contrast, 95% of nurses in a study in Iran were aware of the reporting system, with 60% of participants having used the system [23]. Similarly, 77% of nurses in the study in Malta had completed an incident report, and 72% were aware of where to locate the form [3]. Moreover, senior nurses had a greater understanding of and more experience with reporting incidents [21].

Nurses reported that near-misses were underreported, with some nurses not believing that near-misses are errors [21]. Some nurses were unaware that they made near-miss errors, did not recognise the importance of these errors, did not feel an urgent need to address them, and did not report them [30]. Near-miss errors are seen as routine, a part of the job, and natural mistakes [30]. However, 41% of nurses in a study in Taiwan declared that they voluntarily reported near-misses [27].

### 4.8. Education and Training on Reporting

The theme “Education and training on reporting” explains the influence of education and training on incident reporting and how a lack of education and training was identified as a barrier to incident reporting.

Mansouri et al. [4] found that a lack of education on the error reporting process led to barriers to reporting. Additionally, Abdelmaksoud et al. [21] reported a lack of understanding and familiarity with the reporting system as a barrier to reporting. Similarly, Majda et al. [7] found that a lack of understanding about the complicated paperwork was a barrier to reporting.

The lack of education and training led to a lack of definition of an error [28] and a poorly defined description of what constitutes an error [4]. This also created a lack of agreement over what errors should be reported [4], with some senior nurses advising that there was no need to report the error [30] or that errors were not deemed severe enough to report [4]. This lack of clarity, with no criteria for error severity, unclear responsibility on who is to report, and unclear to whom nurses need to make the report, can create tensions and barriers to reporting [30]. Mrayyan et al. [32] report that education about errors, focusing on defining and agreeing on what constitutes a medication error and reporting such errors, would support incident reporting.

### 4.9. Benefits of Reporting

The theme “Benefits of reporting” describes the benefits, as perceived by nurses, of reporting incidents. Nurses describe learning from errors and improvements to patient safety, but are sceptical about the advantages of near-miss reporting.

In the qualitative study by Abdelmaksoud et al. [21], the benefits of incident reporting were reported as staff learning from errors, education and awareness about errors, looking for trends, addressing system weaknesses, and overhauling systems to avoid similar events. Nurses in Poland thought reporting adverse events was essential and valuable [7]. Specifically, over 80% of participants felt that patient safety is improved by reporting adverse events, and 50–60% thought proactive action may result from error reporting [7]. Similarly, in the quantitative study by Napoli [8], 80% of respondents recognised the helpfulness of reporting errors. Participants of Abdelmaksoud et al. [21] had the following to say about the benefits of reporting: “I suppose the reporting and that allows us to identify if there’s a trend, and if there is a trend, you know, do we [do....] education action plans” and “You cannot learn from your mistakes if you do not report”.

Some nurses felt sceptical about how near-miss reporting could circumvent existing errors [30]. They wondered about the effects of near-miss reporting, as no actions like timely feedback occurred, and they felt that such reporting was meaningless [30]. This eventually resulted in nurses having negative perceptions of near-miss reporting, as it seemed like these reports were brushed aside [30].

### 4.10. Changing the Culture

The theme “Changing culture” explains the strategies to shift from a blame culture to one of enablement. These strategies included patient safety training and open and transparent support from management to change behaviour.

The study by Alsulami et al. [25] illustrated high reporting rates attributed to a pharmacy awareness campaign, patient safety courses, and safety reporting systems campaigns. They found that incident reporting rises after these programs, and reporting continues to increase yearly [25]. Respondents to Abdelmaksoud et al. [21] noted that user-friendly reporting software and ongoing and repeated training and education on the reporting software were enablers of reporting. Similarly, Napoli [8] found that 81% of responding nurses who had participated in a patient safety training course admitted the value of reporting their errors irrespective of patient outcomes. Respondents who received training in the study by Shemsu et al. [2] were almost three times more likely to submit incident reports than those who did not participate in the training.

Perceiving the support of managers had a statistically significant favourable influence on nurses who reported every or most of the adverse events they encountered [8]. Shemsu et al. [2] found that open communication, support from management, and error report feedback were significantly correlated with enhanced error reporting behaviour.

## 5. Discussions

This review aimed to examine the experiences and perceptions of registered nurses who work in acute care regarding incident reporting. Overwhelmingly, this review identifies that registered nurses are fearful of incident reporting and the potential consequences they may face as a result [4,23,26,28,29,31,33]. The fear of damage to their social standing [28], professional reputation [33], financial security [8,28,31,33], or possible legal action [23,26,29] was a dominant factor in this review. Organisations must work with nurses to support them during errors and near-miss events to enhance incident reporting, overcome this feeling of fear, and foster a just culture.

The blame culture that is present in all levels of leadership structure in clinical practice in healthcare [21,23], where error analysis is used to assign blame to individuals [32,35], presents a significant challenge for nurses and error reporting. This blame culture needs to be replaced with a just and informed culture where all errors are reported, learning occurs, and outcomes are not held against the nurses involved [35]. A just culture where nurses perceive the support of managers will positively influence nurses to report every or most of the adverse events they encounter [8]. This just culture needs to be supported by open communication, support from management, and error report feedback [2]. Organisations must be conscious of their role in promoting incident reporting practices in their health services. It is recommended that hospital administrators foster and role model a just culture that promotes patient safety, open and transparent communication, and continuous learning [23].

This review suggests that nurses perceive the role of education and training in supporting error reporting as significant. Data indicate that education and training focusing on patient safety can increase the incident reporting rate [2,25,32], assisting with future patient safety initiatives. Education and training programs were seen as useful [8] in improving knowledge of what constitutes an error [31], what errors require reporting, and the reporting systems and processes [21]. Additionally, education and training that outline the valuable impact of error reporting on patient safety would enhance voluntary incident reporting [7]. It is promising to note the success stories of changing nurses’ perceptions of incident reporting for the better through education and training. Future research is needed to determine the most effective education and training strategies for improving nurses’ perceptions and submission of incident reports.

A notable aspect of this review is the underreporting of near-miss incidents, which is a persistent issue. Many nurses reported that near-misses were not errors and did not require reporting [21]. Additionally, it was noted that reports of near-miss events did not receive follow-up or feedback from management, so reporting these was perceived as futile [30]. Reporting near-miss events is critical to ensure these events are eliminated before harm occurs. Interventions to improve incident and adverse event reporting, such as creating a just culture and providing education and training, will also increase near-miss reporting [36]. Of note, the ability for healthcare providers to report anonymously has been shown to increase near-miss reporting in the literature [35,36,37,38]. To support reporting of near-miss events, Small et al. [35] report providing indemnity for the report maker along with easy systems and processes with practical and accessible feedback to the reporting discipline. Additionally, management must equate near-miss reporting with incident reporting to allocate a similar status and value so that near-miss reporting is seen as a value add to patient safety.

Patient safety incidents can negatively impact nurses’ emotional well-being, resulting in them becoming the second victim [39]. Nursing administrators and healthcare organisations should consider the adverse psychological effects nurses can experience following these events [39]. Providing emotional support, a non-punitive approach, and a safe, comfortable work environment for nurses can help lessen the secondary harm they experience and promote a positive safety culture [39].

### Strengths and Limitations

The strengths of this scoping review lie in the adherence to systematic processes and reporting guidelines. The comprehensive search strategy conducted by two researchers across two databases adds rigour to the findings. Additionally, the screening conducted independently by two researchers minimised selection bias. The data extraction table was piloted and revised to ensure that the data extracted from the included papers would align with the aim of the scoping review. Supporting the generalizability of findings is that the contexts of all papers included in the review relate to nurses in acute care. However, the diversity of healthcare organisations, the nurses who work in them, and cultural elements may constrain this.

However, this review has limitations. The review was limited to English, which may have excluded relevant papers published in other languages. Despite a thorough search strategy, there is a risk that some studies were missed. The review may be limited due to the geographical regions where studies are published. The diverse international settings of the reviewed studies will have variances in regional trends and local health governance, which can influence reporting behaviours and limit the transferability of the findings. For example, Alabdullah and Karwowski [40] examined patient safety culture across continents and found that Africa showed the lowest average patient safety culture scores, emphasising a demand for targeted interventions. However, a common weakness globally was non-punitive error responses and lower positive views on communication openness reported by nurses [40]. Additionally, as 76% of studies in this literature review were quantitative, using surveys to gather results, not all nurses’ unique experiences with incident reporting may be captured. Moreover, most quantitative study designs used retrospective data collection, relying on respondents’ memories of events prior to the survey, potentially resulting in recall bias. Therefore, it is questionable to what extent appropriate conclusions can be drawn based on this scoping review since the surveys were conducted in different countries and are retrospective in nature. The importance of the topic is not in doubt, but a planned, prospective study follow-up, where the health consequences caused by incidents are also evaluated, would be useful. The publications reviewed do not provide sufficient concrete evidence on the process of concealing malpractice and the chances of its effective prevention. Finally, as bedside nurses were the focus of this scoping review, a better understanding of incident reporting behaviours would be gained if reviews focusing on other staff in the healthcare team were conducted.

## 6. Conclusions

This literature review examined the experiences of acute care nurses with incident reporting. Overwhelmingly, nurses reported experiencing fear of incident reporting. Organisational blame culture was identified as a significant barrier to reporting. Furthermore, nurses’ lack of knowledge on what and how to report incidents and how reporting can influence patient safety was identified.

To enhance incident reporting, promoting a just culture that supports and guides nurses who experience near-misses and errors is necessary. Perceiving the support of managers, open communication, and receiving feedback on reported errors and near-misses were significantly associated with incident reporting behaviour. Education and training programs that include the importance of near-miss reporting are recommended. Future research is needed on what education and training strategies are most effective in improving nurses’ perceptions of incident reporting. Changing the culture of fear to one of learning is not optional—it is essential to improving patient outcomes.

## Figures and Tables

**Table 1:** Search strings.

Search ID#	Search Terms	Search Options
S1	TI nurs* OR AB nurs*	Search modes—Proximity
S2	(MH “Nursing Staff, Hospital”)	Search modes—Proximity
S3	S1 OR S2	Search modes—Proximity
S4	TI (Experience* OR perception* OR attitude* OR view* OR feeling* OR perspective*) OR AB (Experience* OR perception* OR attitude* OR view* OR feeling* OR perspective*)	Search modes—Proximity
S5	TI (((incident* OR error* OR “near-miss*” OR “adverse event*”) N2 report*)) OR AB (((incident* OR error* OR “near-miss*” OR “adverse event*”) N2 report*))	Search modes—Proximity
S6	(MH “Incident Reports”)	Search modes—Proximity
S7	S5 OR S6	Search modes—Proximity
S8	TI (hospital* OR (acute N2 care)) OR AB (hospital* OR (acute N2 care))	Search modes—Proximity
S9	(MH “Hospitals+”)	Search modes—Proximity
S10	S8 OR S9	Search modes—Proximity
S11	S3 AND S4 AND S7 AND S10	Limiters—Publication Date: 20190101-20241231 Narrow by Language—English Search modes—Proximity

* This is a truncation symbol, allowing you to find different word endings of a keyword when searching databases.

**Table 2:** Characteristics of included studies.

Author and Year	Study Design	RESEARCH Aim/Question	Location andSetting	Participants	Findings	Other Findings				JBI Quality Appraisal
					Experience and Perceptions of Reporting	Reporting Practices	Barriers to Reporting	Enablers to Reporting	Recommendation	
Abdelmaksoud et al. [21].	Qualitative interview study.	Evaluate current Medication Error Reporting practices and attitudes.	Australian regional hospital.	Twelve participants with 6 nurses.	Clinicians describe the benefits of reporting: staff learning from errors, education and awareness about errors, looking for trends and addressing those system weaknesses, and overhauling systems to avoid similar events. Participants said, “I suppose the reporting and that allows us to identify if there’s a trend, and if there is a trend, you know, do we [do….] education action plans” and “You cannot learn from your mistakes if you do not report”.	Senior nurses had a higher understanding and more experience in reporting incidents. The seriousness of an error was the determining factor for lodging an incident report. Reportable error definition and error reporting practices were inconsistent. Just two clinicians reported any errors in the last year. All respondents thought near-misses were underreported, and some did not believe that near-misses were errors.	Barriers reported included poor understanding of the reporting system, workload pressures, staff shortages, fear of repercussions, and a poor culture of the organisation.	Enablers to reporting included user-friendly reporting software, ongoing and repeated training, education, and peer support.	To support incident reporting, the following options were proposed: Protected time to report promptly, mentoring by experienced staff, more feedback on the incident reports, and a non-punitive approach. Understanding nurses’ motivations, attitudes, and beliefs is important to understanding the culture and incident reporting.	80% Lockwood et al. [22].
Al-Oweidat et al. [23].	Quantitative cross-sectional study.	To develop insight into factors that influence nurses’ willingness to report patient safety incidents.	15 Jordanian hospitals.	325 nurses responded.	Discrepancies exist between the intention to report incidents and actually reporting incidents. Respondents were most likely to report when a patient received the wrong treatment or procedure. Respondents were least likely to report breaches in confidentiality.	Three hundred eight nurses were aware of the reporting system, with 196 having used the system.	The most significant barrier to incident reporting was worry about disciplinary action. Other barriers include concern about getting into trouble, no feedback received on past reports, perceiving that no report is necessary if the incident was discussed with the person involved, unease about who else can see the information disclosed on reports, not feeling responsible for reporting others’ mistakes, unsupportive coworkers, and fear of litigation.	Positive leadership behaviours enhance incident reporting practices (*p* < 0.001). Positive organisational culture also enhances incident reporting practices (*p* < 0.001).	Develop nurse leaders who will generate a supportive and just culture to enhance nurses’ incident reporting practices. A culture that values and supports reporting incidents without fear of retribution.	83% Moola et al. [24].
Alsulami et al. [25].	Quantitative cross-sectional study.	Assess the knowledge, attitudes, and practices of nurses and physicians on medication error reporting.	Saudi Arabia hospital.	365 participants with 303 nurses.	Being a non-Saudi nurse was a significant factor associated with a more favourable attitude towards reporting medication errors (*p* < 0.05).	Nurses favour reporting regardless of the seriousness of the condition.		This study had high reporting rates (55.2%). They attribute this to a pharmacy awareness campaign, patient safety courses, and safety reporting systems campaigns; incident reporting rises yearly after these programmes.	They recommend the establishment of compulsory medication safety courses.	83% Moola et al. [24].
Bany Hamdan et al. [26].	Quantitative cross-sectional study.	Investigate oncology staff’s attitudes, perceived barriers, and strategy toward reporting incidents and errors in the oncology setting.	Saudi Arabia hospital oncology setting.	211 respondents with 139 nurses.	Respondents felt a need to reveal errors and indicated that they would not hide or deny reporting an error in self-interest. A total of 62% (*n* = 92) of nurses disagreed with the statements that admitting to an error would make them feel like a failure or affect their self-esteem. A total of 22% (*n* = 31) indicated that those who make an error are subjected to humiliation or blamed by their colleagues 25% (*n* = 34).	A total of 68% of nurses agreed to report errors.	A total of 64% (*n* = 89) of nurses are worried about legal or disciplinary 55% (*n* = 76) action. A total of 63% (*n* = 87) did not want to get into trouble. A total of 44% (*n* = 62) reported unsupportive colleagues, and 49% (*n* = 68) did not want to be blamed unfairly for the event. A total of 30% (*n* = 43) indicated that the incident report takes too long. Confidentiality in the report information was also reported as a barrier for 35% (*n* = 49), with 32% (*n* = 49) nurses indicating they did not know who had access to the reports.	Nurses want clear guidelines about indent reporting, the ability to report anonymously, and mentors who support and encourage reporting. Clarification about confidentiality and feedback about the incident were also ranked highly.		83% Moola et al. [24].
Chiang et al. [27].	Quantitative cross-sectional survey.	This study examines incident reporting culture and perception of voluntariness incident reporting.	6 teaching hospitals in southern Taiwan.	1380 nurses.	A total of 48.8% and 41.2% of the nurses were committed to voluntarily reporting errors and near-misses, respectively.	A total of 60% of nurses admitted low voluntary incident reporting.		Reporting culture, nursing safety practices, and job satisfaction positively influence voluntary incident reporting.	They recommend a positive reporting culture emphasised by a system-driven, blame-free, learning-based framework. Openly recognise and reward nurses who contribute to patient safety improvement through incident reporting systems. Additional investigation of the incident reporting culture.	83%Moola et al. [24].
Ghobadian et al. [28].	Qualitative semi-structured interviews and focus groups.	Identifying the barriers to reporting clinical errors in the operating theatre and the intensive care unit.	Iranian university hospital ICU and operating theatre setting.	Thirty nurses and 15 physicians were interviewed, and 12 participants were included in a focus group.	A natural inclination is to cover up errors to maintain one’s social status. Nurses experience fear that they are risking their job security when reporting an error.		There is a lack of education and training and no definition of an error. Incident reporting is time-consuming, and wards are often understaffed. Nurses fear they will be blamed, and coworker support is lacking. Nurses also suggest blame from management and the lack of governance as other barriers to reporting. Finally, the lack of feedback on the incident report and the inactive participation of managers were also barriers.	Creating the necessary incentives for nurses to report clinical errors.		80% Lockwood et al. [22].
Kapil and Anoopjit [29].	Quantitative using cross-sectional survey.		Two acute care hospitals in Ludhiana, India.	60 staff nurses.	A total of 48 (80%) had a positive attitude towards incident reporting.	A total of 35 (58.33) had average knowledge of reporting practice.	Fear of legal action 47 (78.33%) and too busy/lack of time 43 (71.66%) were common barriers staff nurses perceive regarding incident reporting.			
Lee [30].	Qualitative interview study.	Describe experiences and perceptions of persons involved in near-miss error reporting omissions.	South Korea hospitals.	9 nurses.	Nurses are sceptical about how near-miss reporting will contribute to the prevention of actual errors. Nurses doubt the effects of near-miss reporting as no timely feedback occurred. Nurses who entered detailed near-miss reports and received no feedback or follow-up after the report felt that such reporting was meaningless. Negative impressions on near-miss reporting as it is brushed aside. Nurses report feeling scared, burdened, and alone when near-miss events happen. Nurses have a fear of miscommunication when raising the event and of being perceived as incompetent.	Nurses were unaware that they made near-miss errors, did not recognise the seriousness of these errors, and did not report them. Near-miss errors are seen as routine, a part of the job, and natural mistakes.	Lack of knowledge, and there are no clear criteria for error severity. The senior nurse advises that there is no need to report a near-miss error. Nurses are unclear about whose responsibility to report the incident and unsure to whom they need to make the report.		Sharing the process and results of near-miss errors. Reframe near-miss errors and view them as opportunities to enhance nursing practice. Supplementary training to raise awareness about the nature and importance of near-miss errors. Education about near-miss errors should be included in the curriculum for undergraduate nurses.	80% Lockwood et al. [22].
Mahdaviazad et al. [31].	Quantitative cross-sectional study.	Evaluate key aspects of medical errors for healthcare providers.	Iran hospital.	164 participants, of which 77 were nurses.	Nurses are more likely to witness an error (*p* = 0.03) and would only report colleagues in the case of a significant incident (32%). The number of reports is significantly less than the number of incidents witnessed.	Nurses are more likely to undertake formal incident reporting (<0.001) than physicians. Nurses were more familiar with the definition, classification, and identification of medical errors (*p* < 0.001) than physicians. A total of 10.8% of nurses reported having good knowledge regarding adverse events. Nurses with more work experience reported higher levels of knowledge (*p* < 0.001).	Fear of litigation, anxiety about being blamed, and receiving punitive action. Lack of anonymity is also a barrier for nurses.			83% Moola et al. [24].
Majda et al. [7].	Quantitative cross-sectional survey.		9 hospitals in Poland.			Nurses thought reporting adverse events was essential and valuable. >80% of nurses felt that reporting adverse events improves patient safety, and 50–60% thought it may result in preventative actions.			Anonymous reporting options. Analysing the reported adverse event to improve patient safety rather than blaming reporters.	83% Moola et al. [24].
Mansouri et al. [4].	Quantitative cross-sectional survey.	Determining the main barriers to reporting errors and adverse events from the standpoint of nurses working in critical care.	7 Iranian hospitals.	251 nurses.	Nurses experienced fear of the impact of reporting an error.	A total of 49% of nurses had experienced an error; however, 71% had not reported it.	Worry of blame and punitive action, demotion, financial penalties, and no support from leaders. There is no training on the reporting process and no clear description of an error. Blame culture instead of patient safety culture, no feedback.		An easy and affordable error-recording system and implementing educational classes are recommended. Effective rapport between managers and nurses will ensure that staff feel safe in reporting any errors.	83% Moola et al. [24].
Mrayyan et al. [32].	Quantitative cross-sectional design.	What are the differences between RNs’ views on reporting medication errors among small, medium, and large-sized hospitals in Jordan?	Four hospitals in Jordan.	229 registered nurses.		A total of 52.8% of nurses were confident about what constitutes medication errors, and 51.1% were sure when to report medication errors using incident reports. A total of 51.5% of nurses considered medication errors should be “serious” to be reported.	A total of 0.3% of incidents are not reported because they fear disciplinary actions or losing their jobs. (59.8%) are not reported because of the fear of the reactions of their coworkers, and 57.6% are not reported because of fear of the nurse managers and administrators’ reactions.	A standard incident report form that is easy to use and should contain questions that can assist in categorising medication errors must be available in all hospital departments. In-service education about medication errors is needed to define and agree on what constitutes a medication error and how to report such errors. Rather than disciplining nurses who commit mistakes, awareness that reporting errors is necessary to modify the hospital system to prevent future errors.		
Napoli [8].	Quantitative exploratory survey.	Research the perceptions of nurses regarding incident reporting systems.	Eastern Italy hospital.	122 nurses.	Nurses reported experiencing fear when reporting an incident. Other feelings, such as mistrust, resignation, and scepticism, were also reported. A total of 80% saw value in reporting their errors.	A total of 43% (*n* = 53) of nurses rarely reported an event, while 14% (*n* = 17) reported any event witnessed. Only 40% were aware of the online reporting system for incidents.	Barriers were identified as fear of consequences and lack of time available.	Support of leadership has a significant positive influence on reporting adverse events. A total of 81% of nurses who had patient safety training valued reporting.	Feedback on improvement interventions after sending incident reports is important to increase willingness to report.	83% Moola et al. [24].
Rashed and Hamdan [33].	Quantitative cross-sectional survey.	Assess the attitudes of physicians and nurses toward incident reporting in Palestinian hospitals.	11 hospitals in Palestine.	475 participants, with 323 nurses.	Nurses feel that supervisors support those who report errors. Nurses experienced fear of incident reporting, concerned their competence would be questioned, and loss of colleagues’ respect.	There is a high level of awareness among nurses to report; however, physicians were 2.1 times more likely to report incidents than nurses.	The highest barriers to reporting were lack of feedback about the medical errors, reporting pinpoint blame and lack of supervisor support. Other barriers included fear of sanctions, litigation, and revenge from patients or families.	Improving patient safety, learning from errors, and preventing future errors.	Voluntary reporting systems that support confidentiality with clear definitions of what should be reported.	83% Moola et al. [24].
Shemsu et al. [2].	Quantitative cross-sectional study design.	Assess patient safety incident reporting behaviour and its associated factors among healthcare professionals.	4 public hospitals in Ethiopia.	334 participants with 205 nurses.		A total of 37% of nurses reported yes to incident reporting behaviours. A total of 64.7 % were unclear about their role in the incident reporting. Over one-third do not know how to obtain an incident form or where to lodge it.	Administrative sanction was a barrier to reporting.	Open communication, support from leadership, and feedback on errors and management support are associated with incident reporting behaviours. Trained staff were nearly 3 times more likely to lodge incident reports.	On-job training related to patient safety incidents should be provided, including open discussion on the purpose and goals of patient safety incident reporting. A culture that fosters teamwork is needed to support incident reporting behaviours. A well-established incident reporting system for patient safety should be used.	83% Moola et al. [24].
Ward and Mangion [3].	Quantitative cross-sectional design.	Assess the nurses’ attitudes and practices of incident reporting.	Malta’s acute general hospital.	323 nurses.	Nurses reported feeling stressed that people would turn against them, lacking trust in the organisation, and being disappointed about not receiving feedback.	A total of 77% of nurses had completed an incident report. A total of 72% (*n* = 232) were aware of where to locate the form.	No feedback on incident reports. Near-misses not viewed as valuable to report. Views that adverse event reporting will not lead to system changes that will improve the quality of care.			83% Moola et al. [24].

## Data Availability

The original data presented in the study are openly available online.
